# Impairment in dynein-mediated nuclear translocation by BICD2 C-terminal truncation leads to neuronal migration defect and human brain malformation

**DOI:** 10.1186/s40478-020-00971-0

**Published:** 2020-07-14

**Authors:** Meng-Han Tsai, Haw-Yuan Cheng, Fang-Shin Nian, Chen Liu, Nian-Hsin Chao, Kuo-Liang Chiang, Shu-Fang Chen, Jin-Wu Tsai

**Affiliations:** 1 Department of Neurology, Kaohsiung Chang Gung Memorial Hospital, Kaohsiung, Taiwan; 2 School of Medicine, College of Medicine, Chang Gung University, Taoyuan, Taiwan; 3 Institute of Brain Science, National Yang-Ming University, No.155, Sec.2, Linong Street, Taipei, 112 Taiwan; 4 Program in Molecular Medicine, National Yang-Ming University and Academia Sinica, Taipei, Taiwan; 5 Department of Pediatric, Kuang Tien General Hospital, Taichung, Taiwan; 6 Brain Research Center, National Yang-Ming University, No.155, Sec.2, Linong Street, Taipei, 112 Taiwan; 7 Biophotonics and Molecular Imaging Research Center, National Yang-Ming University, No.155, Sec.2, Linong Street, Taipei, 112 Taiwan; 8 Department of Biological Science & Technology, National Chiao Tung University, Hsin-Chu, Taiwan

## Abstract

During brain development, the nucleus of migrating neurons follows the centrosome and translocates into the leading process. Defects in these migratory events, which affect neuronal migration, cause lissencephaly and other neurodevelopmental disorders. However, the mechanism of nuclear translocation remains elusive. Using whole exome sequencing (WES), we identified a novel nonsense *BICD2* variant p.(Lys775Ter) (K775X) from a lissencephaly patient. Interestingly, most *BICD2* missense variants have been associated with human spinal muscular atrophy (SMA) without obvious brain malformations. By in utero electroporation, we showed that BicD2 knockdown in mouse embryos inhibited neuronal migration. Surprisingly, we observed severe blockage of neuronal migration in cells overexpressing K775X but not in those expressing wild-type BicD2 or SMA-associated missense variants. The centrosome of the mutant was, on average, positioned farther away from the nucleus, indicating a failure in nuclear translocation without affecting the centrosome movement. Furthermore, BicD2 localized at the nuclear envelope (NE) through its interaction with NE protein Nesprin-2. K775X variant disrupted this interaction and further interrupted the NE recruitment of BicD2 and dynein. Remarkably, fusion of BicD2-K775X with NE-localizing domain KASH resumed neuronal migration. Our results underscore impaired nuclear translocation during neuronal migration as an important pathomechanism of lissencephaly.

## Introduction

Nuclear migration is important to many forms of cellular behavior. Typically, nuclear movement is mediated through tightly regulated forces exerted on the cytoskeleton by molecular motors [5, 12–14, 30]. The development of the vertebrate central nervous system (CNS) involves a particularly important and complex series of migratory events dependent on nuclear migration over large distances at many stages of development. In the developing cerebral cortex, cortical neurons are born in the ventricular zone (VZ) and migrate over substantial distances to form the highly organized cortical layers [6, 33]. Postmitotic neurons extend a leading process and migrate in a “two stroke” manner. The centrosome first departs from the nucleus and moves into a dilated region of the leading process; the nucleus then funnels through the leading process and catches up with the centrosome [2, 39, 40, 48]. This process, termed “nuclear translocation”, requires cytoplasmic dynein and its regulator LIS1, as well as non-muscle myosin II [41, 54].

Severe impairments in neuronal migration during brain development lead to the neurodevelopmental disorder lissencephaly, characterized by smooth cerebral surface, absent or decreased gyri, thickened cortex and enlarged ventricles [1, 16]. Lissencephaly patients suffer from epilepsy, hypotonia, mental retardation and developmental delay [11, 16]. Pathogenic variants in genes that encode the components and regulators of dynein and microtubules, such as *PAFAH1B1* (also known as *LIS1*), *DCX*, *TUBA1A*, are the major genetic causes of this devastating disease [11, 16]. By live imaging of migrating embryonic rat neurons, LIS1 was shown to assist dynein within the leading process to pull the centrosome-centered microtubule network [48]. Based on the organization of the microtubules, the nuclei appear to bind to the microtubules and translocate toward their minus ends at the centrosome, similar to the retrograde transport of organelles by cytoplasmic dynein. However, how dynein is recruited to the nuclei of migrating neurons remains poorly understood.

Recently, BICD2 (Bicaudal D Homolog 2), the homolog of *Drosophila* Bicaudal D [19, 20], has been implicated in nuclear migration during a variety of cellular behaviors. BICD2 was found to associate with a component of nuclear pore complexes (NPCs), RanBP2, and recruit dynein-dynactin to tether centrosomes to the nuclei prior to mitotic entry [42, 43]. The RanBP2-BicD2 pathway is also essential for the apical nuclear migration in radial glial cells (RGCs) during G2 phase of the cell cycle in developing rat brains [22]. BicD2-null mice exhibited an enlarged ventricle and disrupted laminar organization of cerebral cortex and the cerebellum, which suggests that BicD2 is essential for normal brain development [24]. Neuron-specific ablation of BicD2 also led to defects in radial migration of upper-layer neurons [56]. Interestingly, heterozygous missense variants in human *BICD2* cause autosomal dominant lower extremity-predominant spinal muscular atrophy 2 (SMALED2; MIM # 615299), which presents a loss of spinal motor neurons, muscle weakness, and atrophy predominantly of the lower limbs [31, 34, 35]. However, most patients with heterozygous missense BICD2 variants did not exhibit obvious CNS malformation except two cases of polymicrogyria [36].

Here we identified a novel de novo *BICD2* nonsense variation p.(Lys775Ter) (K775X) from a lissencephaly patient using whole-exome sequencing (WES). Unlike previous *BICD2* variants found in SMALED2 patients, this variant led to a truncated form of BICD2. We showed that expression of BicD2 K775X in the developing mouse brain severely disrupted the radial migration of cortical neurons. This truncated BicD2 mutant failed to localize at the nuclear envelop (NE), and hindered NE recruitment of the dynein complex. We also showed an interaction between Nesprin-2 and BicD2 [15], which was disrupted by the p.(Lys775Ter) variant. Remarkably, fusion of BicD2 K775X with a NE-localizing domain KASH rescued the neuronal migration defect in the developing mouse cortex. Our study reports the first disease-causing *BICD2* nonsense variant in lissencephaly. Moreover, our functional study demonstrated the important roles of BICD2 in neuronal migration [56] and human brain development.

## Results

### WES identified a novel truncated *BICD2* mutation in a patient with both lissencephaly and SMALED

**Fig. 1:**
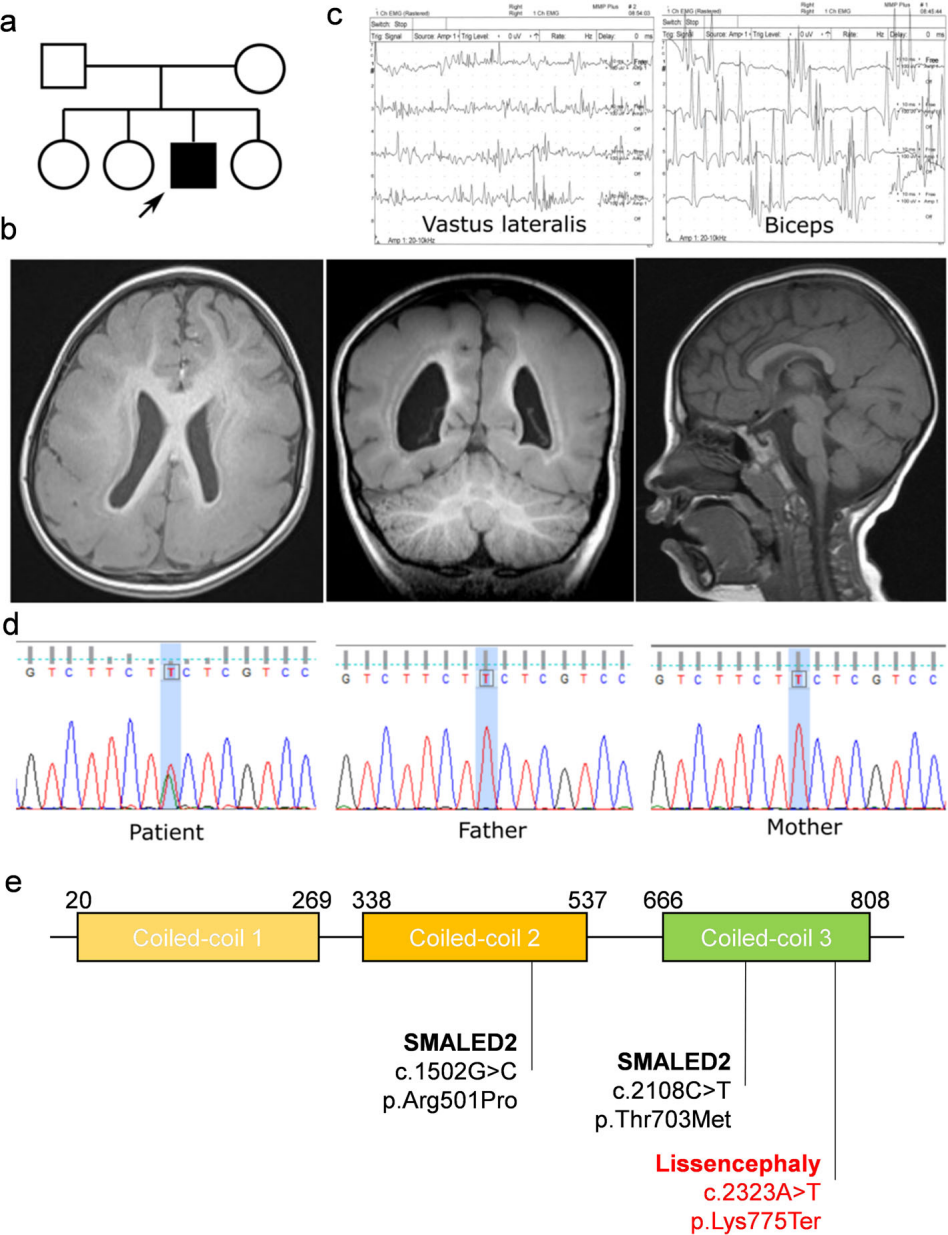
Clinical representation. **a** Pedigree and clinical features of posterior predominant lissencephaly and SBH caused by *BICD2* nonsense mutation. **b** The brain MRI scans showed no obvious brainstem or cerebellar atrophy and preserved corpus callosum. **c** Electromyography findings demonstrated predominant myogenic changes in lower limbs and neurogenic pattern in upper limbs. **d** Chromatography confirmed de novo mutation of *BICD2* gene. **e** The three coiled-coil (CC) domains were illustrated, and the truncated mutation of the proband affects the C-terminal end of CC3 domain

The proband, who is the third child of an unrelated Taiwanese couple, is a 4-year-old boy with global delay in reaching developmental milestones (Fig. 1a). Mild ventriculomegaly was diagnosed at 28 weeks of gestational age by prenatal ultrasound. He was born at term through normal vaginal delivery. Bilateral leg weakness (left > right) was noted at 1 years old because he started to crawl with both hands. After intensive rehabilitation, he started to walk with support and could sit-up alone without support after 2 years old. He could only speak a few simple words (pa-pa, ma-ma) and progressed to walking with a waddling gait without support at the time of assessment (4 years old). He could not follow simple verbal comments but responded to social smiling and used simple gesture clues (pointing and reaching). There was no family history of intellectual disability or seizures. Physical examination revealed microcephaly with a head circumference of 45 cm (< 3rd percentile) and bilateral lower limb weakness (Medical Research Council Scale = 4) with increased spasticity (Ashworth scale = 2). Brain MRI revealed posterior predominant thick lissencephaly with underlying thin layer of subcortical band heterotopia (SBH), but no signs of polymicrogyria or cerebellar atrophy (Fig. 1b). Electromyography showed small motor unit action potential with early recruitment in the lower limb muscles (vastus lateralis, vastus medialis and adductor), which suggests myopathic changes. Mildly reduced recruitment and interference pattern were observed in his upper limbs (biceps) (Fig. 1c). Furthermore, a nerve conduction study showed normal amplitudes and conduction velocities. Lastly, scalp electroencephalography showed intermittent delta slowing over the bilateral occipital area.

WES of the proband resulted in 83,407 variants that were different from the reference genome (hg19). Using in-house controls of 63 WES data processed by the same pipeline as filter, 2944 variants remained that is not seen in any other individuals. The variants were further filtered by the following criteria: located in exons or splice site, not synonymous change, not present in four control databases (TPG, ESP, ExAc, gnomAD) and predicted to be pathogenic by four in-silico computational algorithms (PolyPhen2, PROVEAN, MutationTaster2 and CADD≥20). Four variants remained after filtering and visual inspection of mapped (BAM) file with Integrated Genomics Viewer, including 3 nonsynonymous (all pass the prediction of four in-silico computational algorithms), and 1 stop gain variants (Table 1). Sanger sequencing of the proband and his parents confirmed that only the stop gain variant in *BICD2* gene (NM_015250, c.2323A > T p.(Lys775Ter)) was de novo (Fig. 1d). The variant resulted in a truncation of the C-terminal 81 amino acids (Fig. 1e).

**Table 1:** Candidate variants of WES study after filtering and visual inspection

Chr	Start	Ref	Alt	Gene Name (Ref Seq)	Mutation Type	Amino Acid Change	PROVEAN	Polyphen2	Mutation Taster	CADD	Inheritance
3	44,489,313	C	A	ZNF445 (NM_181489)	Heterozygous Missense	c.G1850T:p.R617M	D	D	D	31	Inherited
8	48,883,365	G	A	MCM4 (NM_005914)	Heterozygous Missense	c.G1729A:p.D577N	D	D	D	33	Inherited
9	95,477,681	T	A	BICD2 (NM_001003800)	Heterozygous Nonsense	c.A2323T:p.K775X	.	.	A	40	De novo
14	20,943,038	T	C	PNP (NM_000270)	Heterozygous Missense	c.T392C:p.L131P	D	D	D	29.2	Inherited

### BicD2 knockdown caused neuronal migration delay during cortical development

**Fig. 2:**
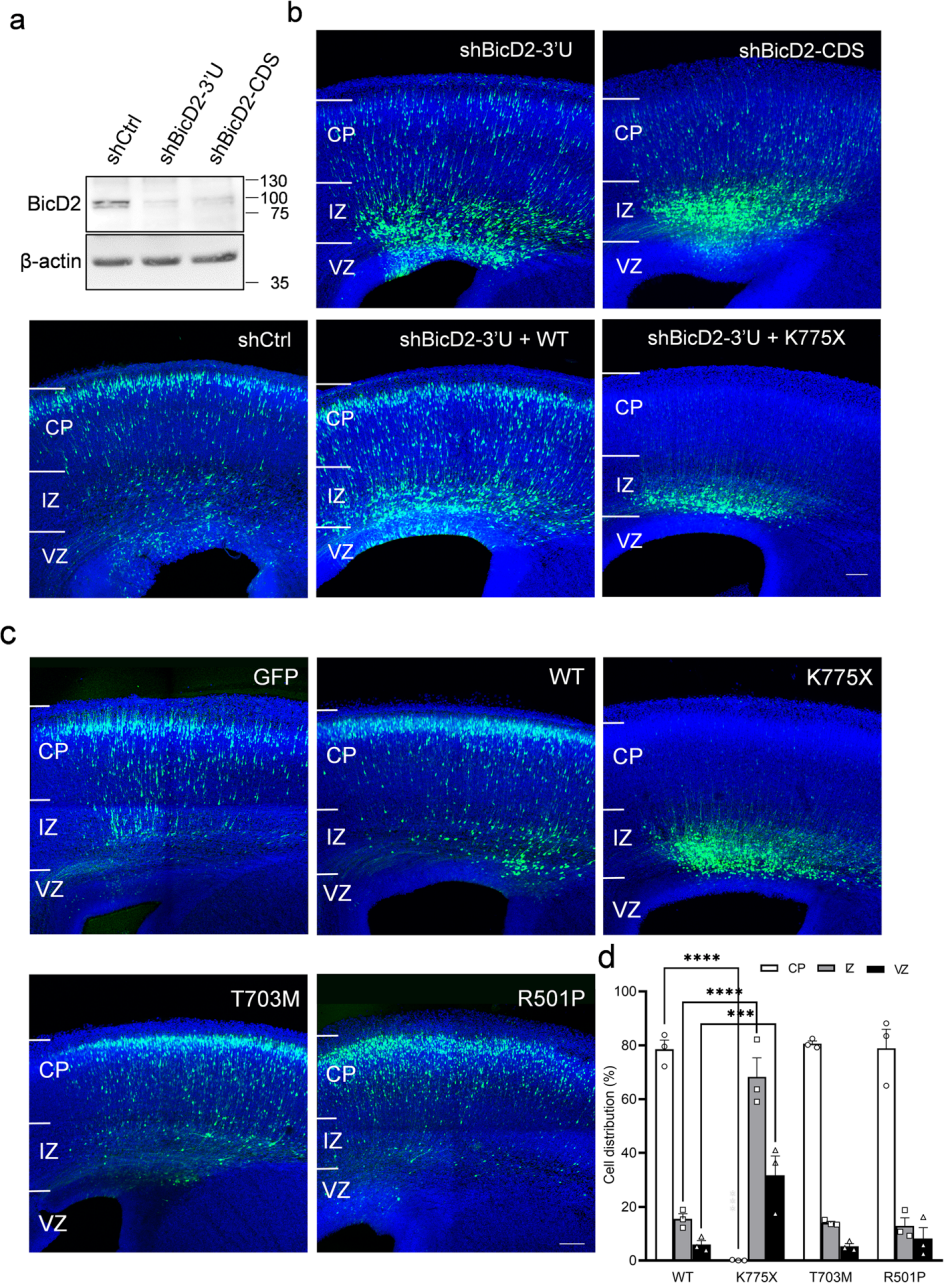
Effects of BicD2 knockdown and mutations on neuronal cell distribution in the neocortex. **a** Western blot of BicD2 in primary neurons infected with lentiviruses encoding BicD2 shRNAs (shBicD2–3’U and shBicD2-CDS). BicD2 expression levels in cells infected with both shBicD2 were much lower than those infected with shCtrl. **b** Cell distributions in the neocortex electroporated with BicD2 shRNA and cDNA. Coronal sections of the mouse brains were collected 4 days after in utero electroporation of indicated constructs along with GFP at E14.5. While most GFP+ cells electroporated with shCtrl had already reached the CP, most cells electroporated with shBicD2-CDS and 3’U were restricted to the VZ and IZ. Expression of WT BicD2 partially rescued the migration defect. Surprisingly, expression of K775X BicD2 led to even more severe neuronal migration impairments. **c** Severe neuronal migration defect by the expression of K775X BicD2. Coronal sections of the mouse brains were collected 4 days after in utero electroporation of BicD2 WT, K775X or SMALED2-associated mutants (T703M and R501P) at E14. Expression of K775X, but not WT or other mutants, caused a severe defect in neuronal migration to the CP. All slices were stained with DAPI (blue) to show the cell nuclei. Bars = 100 μm. **d** Bar graph with individual data points showing the percentage of GFP+ cells in the CP, IZ, and VZ 4 days after electroporation (*n* = 3 pregnant females in each condition). Error bars represent SEM. ***P < 0.001, ****P < 0.0001. ANOVA test. Post hoc: Bonferroni test

Previously, it was shown that BicD2 loss-of-function in the developing rat brain causes defects in neuronal distribution [22]. To study the consequences of BicD2 dysfunctions, we first applied RNA interference (RNAi) to knock down BicD2 expression using 2 shRNAs, each targeting the 3′ untranslated region (3’UTR) and coding DNA sequence (CDS) of mouse BicD2 mRNA (shBicD2–3’U and shBicD2-CDS, respectively). To test the knockdown efficiency of these BicD2 shRNAs, the constructs were packaged into lentiviruses and introduced to cultured cortical neurons isolated from E16.5 embryos. Five days after the viral infection, BicD2 expression in shBicD2-transduced cells was significantly lower (shBicD2–3’U: 13.3 ± 0.03% and shBicD2-CDS: 10.0 ± 0.01%) compared to the control cells infected with lentivirus expressing non-targeting shRNA (shCtrl) (Fig. 2a; Supplementary Figure 1a).

We then introduced shBicD2–3’U, shBicD2-CDS or shCtrl constructs along with GFP cDNA into embryonic mouse brains at E14.5 using in utero electroporation [38, 45, 49], which allows gene transfer to a subset of neural stem cells (i.e., RGCs) in the developing brain. The electroporated mouse brains were then examined 4 days after electroporation. In control brains, most GFP+ cells expressing shCtrl left the VZ and migrated to the cortical plate (CP) (Fig. 2b, Supplementary Fig. 1b), consistent with previous studies [9, 26]. In contrast, brains electroporated with shBicD2–3’U or shBicD2-CDS exhibited altered neuronal distribution, with more than half of the GFP+ cells remaining in the IZ and VZ (Fig. 2b, Supplementary Fig. 1b), indicating a possibility of delayed neuronal migration and/or differentiation. This result was consistent with the previous study using embryonic rat brains [22].

To determine whether the novel p.(Lys775Ter) variant could interfere with the normal function of BicD2 in neuronal migration, we introduced wild type (WT) BicD2 cDNA (referred to as BicD2 WT) or BicD2 p.(Lys775Ter) mutant (referred to as BicD2 K775X) to rescue the delayed migration caused by shBicD2. E14.4 brains were co-electroporated with BicD2 WT or K775X along with shBicD2–3’U, which could not target the BicD2 cDNA due to the lack of 3’UTR in the cDNA. At E18.5, we found that the impairment of neuronal migration could be partially rescued by BicD2 WT but not BicD2 K775X (Fig. 2b, Supplementary Fig. 1b). Surprisingly, the expression of BicD2 K775X appeared to cause a more severe neuronal migration impairment in comparison with brains electroporated with only BicD2 shRNA. We therefore suspected that BicD2 K775X expression may have a dominant-negative effect on neuronal migration.

### BicD2 p.(Lys775Ter) altered neuronal distribution without affecting differentiation

To examine whether BicD2 K775X might have a dominant-negative effect on neuronal migration, we electroporated BicD2 WT or K775X and GFP constructs into embryonic mouse brains at E14.5 (Fig. 2c). Overexpression of BicD2 WT did not cause an apparent abnormality in neuronal distribution 4 days after electroporation. Remarkably, in brains electroporated with BicD2 K775X, most transfected GFP+ cells were arrested in the VZ and IZ (82.2 ± 0.1%, *n* = 3 animals). In addition, we tested the effects of overexpressing two SMALED2-associated missense variants, p.(The703Met) (T703M) and p.(Arg501Pro) (R501P), which did not cause detectable brain structural changes clinically [31, 34]. Interestingly, overexpression of neither BicD2 T703M nor R501P mutant led to an apparent difference in the spatial distributions of neurons compared to the control brain electroporated with BicD2 WT or GFP only (Fig. 2c). Therefore, our results suggested that BicD2 K775X exerted a dominant-negative effect on neuronal migration, which may underlie the pathological mechanism of lissencephaly.

**Fig. 3:**
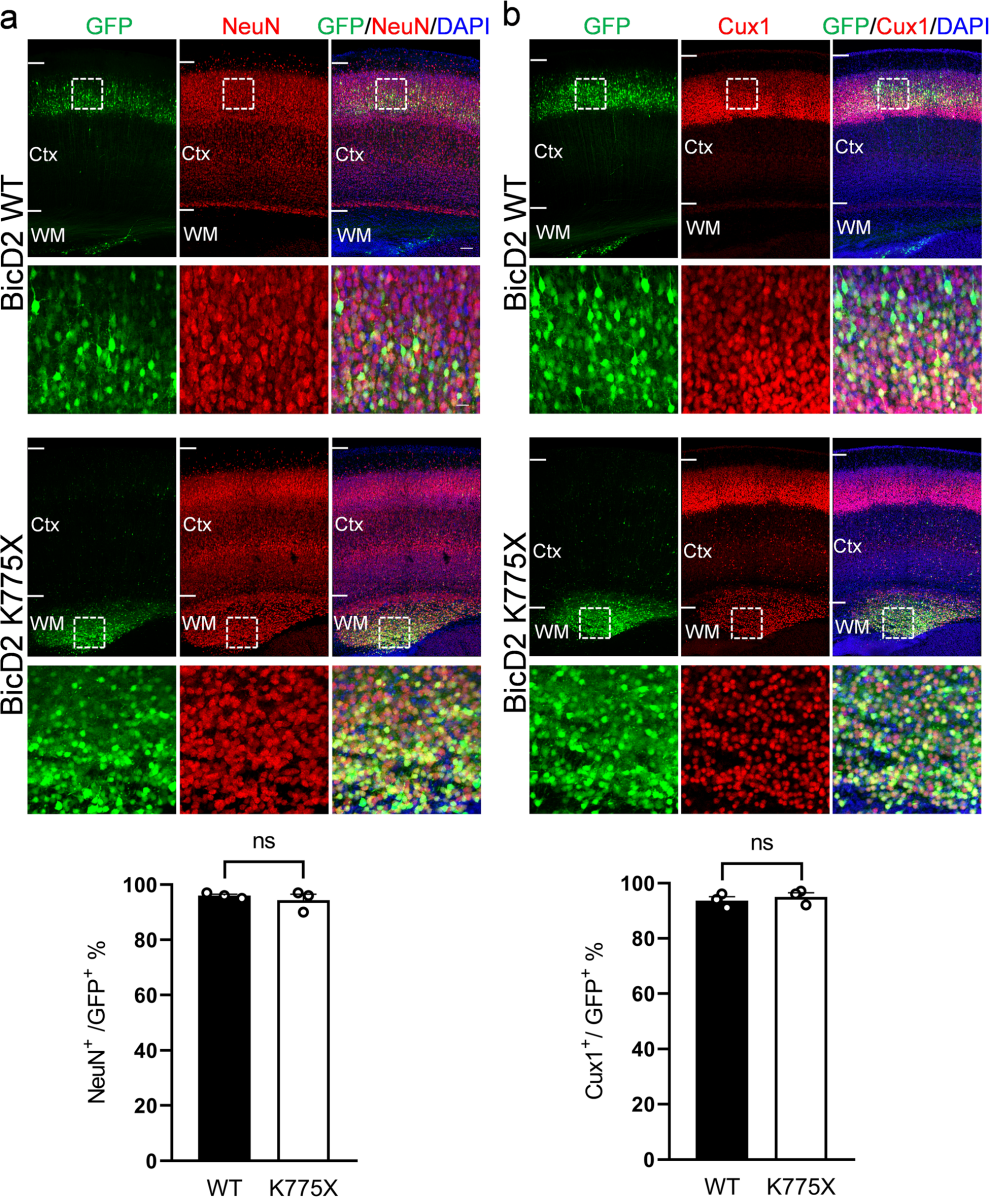
Differentiation of cells expressing BicD2 WT and K775X into cortical neurons postnatally**. a** Expression of neuronal marker NeuN (red) in brains electroporated with BicD2 WT or K775X (green). Coronal sections of the mouse brains were collected at P7 after in utero electroporation at E14.5. The majority of cells electroporated with BicD2 WT or K775X were NeuN+, even though K775X-expressing cells were arrested in the WM. Bar = 100 μm in the top panel, bar = 25 μm in the lower panel. Bar graph with individual data points shows the percentage of NeuN+/GFP+ cells in the electroporated brain slices (*n* = 3 mice from 3 independent pregnancies). Error bars represent SEM. Student’s *t* test. **b** Expression of the marker for cortical layer II-IV, Cux1, in brains electroporated with BicD2 WT or K775X (green). Again, most of the electroporated cells were Cux1+ in both groups. All slices were stained with DAPI (blue) to show the cell nuclei. Boxed regions were shown at a higher magnification below each panel. Bar graph with individual data points shows the percentage of Cux1+/GFP+ cells in the electroporated brain slices (*n* = 3 mice from 3 independent pregnancies). Error bars represent SEM. Student’s *t* test

To examine the fate of these arrested cells, we stained BicD2 WT- and K775X-electroporated brain slices with a variety of markers, including the cell cycle marker Ki67, radial glia cell marker Pax6, intermediate progenitor marker Tbr2, and neuronal marker NeuN (Supplementary Fig. 2). Interestingly, arrested cells in the deeper IZ did not appear to co-localize with Ki67, Pax6, or Tbr2 markers at E18.5, indicating that these cells have exited the cell cycle. However, these cells have not yet started to express NeuN at this stage, similar to control cells [26]. To further explore whether the arrested cells were capable of differentiating into neurons, brain slices obtained at postnatal day 7 (P7) after in utero electroporation at E14.5 were stained with NeuN and the marker for layer II-IV neurons, Cux1 (Fig. 3). Most cells transfected with BicD2 K775X expressed both NeuN and Cux1 (94.33%, Fig. 3a and 95%, Fig. 3b). These results suggested that overexpression of BicD2 K775X in RGCs impaired neuronal migration but not differentiation, similar to previous observations using Lis1 RNAi [49].

### BicD2 K775X impaired neuronal migration and nucleus-centrosome coupling

**Fig. 4:**
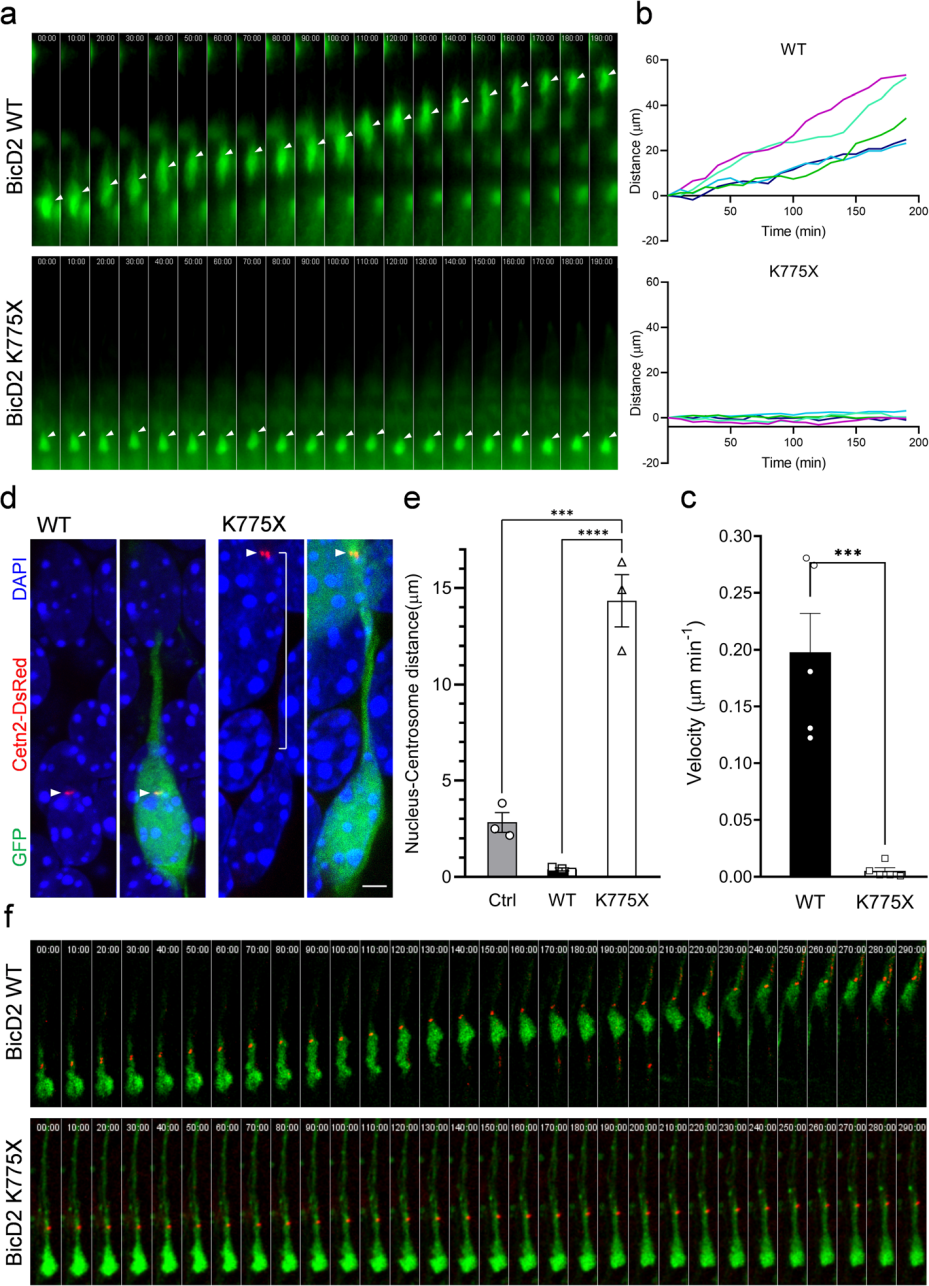
Impaired migration and nucleus-centrosome coupling in neurons expressing BicD2 K775X. **a** Organotypic brain slice cultures from brains electroporated with BicD2 WT or K775X at E14.5 and imaged 2.5 days later. Time lapse images, taken at 10-min interval of a representative cell expressing BicD2 WT, shows continuous migration toward the CP (upper panel). The BicD2 K775X-expressing cell stayed stationary and failed to migrate forward (lower panel). **b** Distance-time graphs of representative cells expressing BicD2 WT (upper panel) showed classic migration pattern, while cells expressing BicD2 K775X (lower panel) showed severe impairment in migration ability. **c** Bar graph with individual data points shows the migration rate of cells expressing BicD2 WT or K775X. Expression of BicD2 K775X a dramatic decrease in migration rate. Error bars represent SEM. ***p < 0.001. Student’s *t* test. **d** Images of the centrosome and nucleus of a migrating neuron electroporated with BicD2 WT or K775X cDNA along with GFP (green) and Centrin II-DsRed (red) at E14.5. Cells were stained with DAPI (blue) to label the nuclei. The centrosomes (arrowhead) in most BicD2 WT-expressing cells were found in the perinuclear area (left panel). In contrast, in cells expressing BicD2 K775X, the centrosomes were often found further along the leading process at a longer distance from the nucleus (right panel). **e** Bar graph with individual data points of the average distance between the nucleus and centrosome in cells expressing GFP, BicD2 WT, and BicD2 K775X (*n* = 3 pregnant females in each condition). Error bars represent SEM. ***P < 0.001, ****P < 0.0001. ANOVA. Post hoc: Bonferroni test. **f** Time lapse images, taken at 10-min interval, of representative cells expressing BicD2 WT or K775X along with Centrin II-DsRed (red) and GFP (green) simultaneously. The cell expressing BicD2 WT showed a classic “two stroke” migration pattern. In contrast, the cell expressing BicD2 K775X remained stationary after the centrosome migrated into the leading process

To directly monitor the effects of BicD2 K775X variant on neuronal migration, we employed time-lapse imaging of neurons in live embryonic brain slices [8, 9, 49]. Brains electroporated with BicD2 WT or K775X at E14.5 were sliced and cultured at E17.5. While control cells electroporated with BicD2 WT cDNA migrated continuously toward the CP, neuronal migration of cells expressing BicD2 K775X was severely disrupted (Fig. 4a-c; Movies S1 and S2). To dissect specific migratory steps that BicD2 may be involved in, we examined nucleus-centrosome (N-C) coupling during neuronal migration [3, 48]. Brains were electroporated with BicD2 WT or K775X cDNA, along with Centrin II-DsRed and GFP at E14.5 and examined at E17.5 (Fig. 4d). Remarkably, the distance between the nucleus and centrosome was significantly larger in cells overexpressing BicD2 K775X than in the cells with BicD2 WT or GFP only (Fig. 4e), suggesting a disruption of N-C coupling during neuronal migration.

We further monitored centrosomal movements in migrating neurons using the time-lapse imaging in brain slices electroporated with Centrin II-DsRed (Fig. 4f). Consistently, cell expressing BicD2 WT performed a classic “two stroke” migration [2, 39, 40, 48]. The centrosome moved into the leading process, followed by nuclear translocation. Instead, cell expressing BicD2 K775X stayed stationary even though the centrosome had moved into the leading process (Fig. 4f).

### K775X disrupted interaction with Nesprin-2 and NE recruitment of dynein and BicD2

**Fig. 5:**
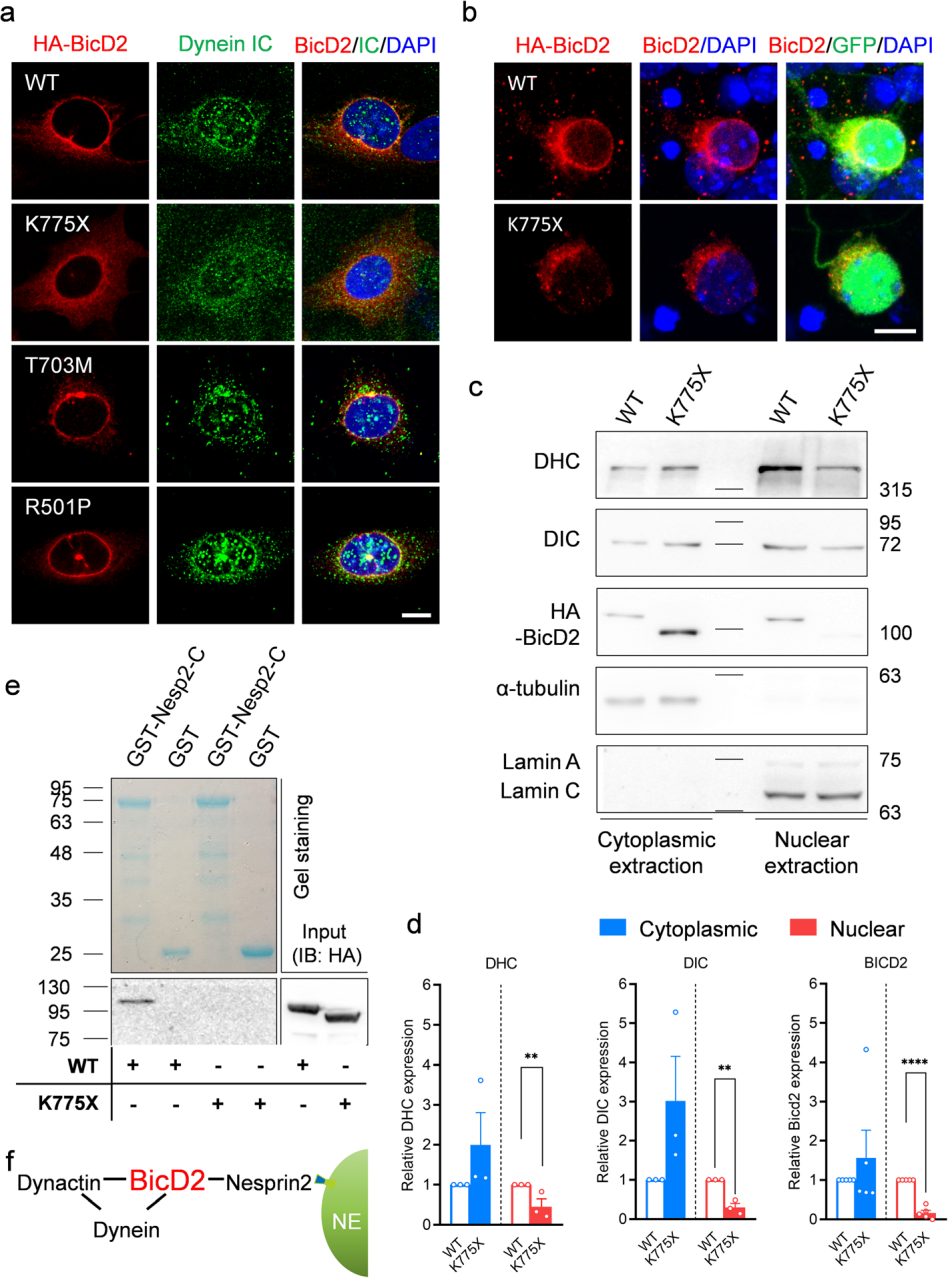
Impaired NE recruitment and Nesprin-2 interaction by BicD2 K775X mutation. **a** Subcellular distributions of BicD2 and dynein in WT and mutant BicD2-transfected cells. U2OS cells were transfected with constructs expressing WT or mutant (K775X, T703M, and R501P) HA-BicD2 proteins and synchronized at G0 phase by serum starvation for 24 h. BicD2 and dynein showed prominent NE distribution revealed by HA and DIC antibodies. BicD2 and dynein were more dispersed in the cytoplasm in cells expressing the K775X mutant, but not the T703M or R501P mutant. Cells were stained with DAPI (blue) to show the cell nuclei. Bar = 5 μm. **b** Subcellular distribution of BicD2 WT or K775X (red) in cultured cortical neurons. E17 neurons were isolated from mouse brains electroporated with BicD2 WT or K775X along with GFP (green) by in utero electroporation at E14. While BicD2 WT mainly localized on the NE, K775X was dispersed in the cytoplasm. Cells were stained with DAPI (blue) to show the cell nuclei. Bar = 5 μm. **c** Western blots of dynein and BicD2 in the nuclear and cytoplasmic fractions from cells expressing BicD2 WT or K775X. K775X mutation leads to decreases in the amount of dynein (judged by both DHC and DIC antibodies) and BicD2 (judged by HA antibody) in the nuclear fraction and increases in the cytosolic fraction. Lamin A/C and α-tubulin were used as the nuclear and cytosolic marker, respectively. **d** Quantification of DHC, DIC and BicD2 in nuclear and cytosolic fractions (*n* > 3 replicated experiments in each groups). Error bars represent SEM. **P < 0.01, ****P < 0.0001. Student’s *t* test. **e** GST pull-down assay to determine the interaction between BicD2 and Nesprin-2. Cell lysate expressing BicD2 WT or K775X were incubated with glutathione-agarose beads preloaded with GST or GST-tagged Nesprin-2 C-terminus (left panel). 2% of the input (right panel) was the positive control. Western blot using HA antibody showed that BicD2 WT but not K775X was pulled down by Nesprin-2 C-terminus. **f** These results suggested a dynein-BicD2-Nesprin-2 link to the NE

The disruption of N-C coupling during neuronal migration by BicD2 dysfunction pointed us to the potential involvement of BicD2 in nuclear translocation, which is mediated largely by the recruitment of dynein motor to the NE [3]. To examine whether BicD2 K775X could affect its localization and NE recruitment of dynein, we used immunofluorescence staining to observe the intracellular distribution of WT and mutant forms of BicD2 in cultured G0 cells (Fig. 5a). Interestingly, while BicD2 WT was predominantly localized at the NE, BicD2 K775X was dispersed in the cytoplasm. In contrast, SMALED2-associated BicD2 variants, T703M and R501P, maintained their ability to localize on the NE. Meanwhile, we also determined the distribution of the dynein complex in these cells by staining the dynein intermediate chain (DIC), which is required for dynein functions [55]. Consistent with the BicD2 distributions, dynein was localized at the NE in cells expressing BicD2 WT, T703M and R501P, while the expression of BicD2 K775X displaced dynein to the cytoplasm (Fig. 5a). To confirm the localization of BicD2 WT and K775X in neurons, these cDNAs along with GFP were electroporated into the brain by in utero electroporation at E14.5. Cortical neurons from E17.5 brains were then cultured and immunostained for BicD2. A clear ring of BicD2 surrounding the nucleus was observed in the neurons expressing BicD2 WT but not K775X, indicating that K775X truncation indeed disrupted NE localization in neurons.

To quantitatively examine the effects of K775X variant on dynein NE localization, we isolated the nuclear and cytosolic fractions of lysates from cultured cells transfected with BicD2 WT or K775X and determined the amount of dynein complex in each fraction (Fig. 5c). In cells expressing BicD2 K775X, the relative amount of dynein heavy chain (DHC) and DIC, as well as BicD2 all significantly decreased in the nuclear fraction compared to those in BicD2 WT-expressing cells (Fig. 5d). This result further confirmed that BicD2 K775X failed to localize to the NE and impaired dynein NE recruitment.

Previous studies suggested that KASH-domain NE proteins Nesprin-1/2 may facilitate NE recruitment of the dynein complex [58]. Very recently, BicD2 has been shown to mediate dynein interaction to NE through its interaction with Nesprin-2 [15]. To examine whether BicD2 K775 variant could impair Nesprin-2-dynein interaction, we used GST pulldown assay to check the interaction of the Nesprin-2 C-terminus with BicD2 WT and K775X. HEK293T cells were transfected with BicD2 WT or K775X cDNA for 24 h, lysed, and incubated with beads conjugated with GST-Nesprin-2 C-terminus. We found that the Nesprin-2 C-terminus could bind to WT BicD2 but not the K775X mutant (Fig. 5e). These results indicated that BicD2 K775X truncation disrupts its interaction with Nesprin-2 and its recruitment of dynein to the NE (Fig. 5f).

### Recruitment of BicD2 K775X to NE rescued neuronal migration delay

**Fig. 6:**
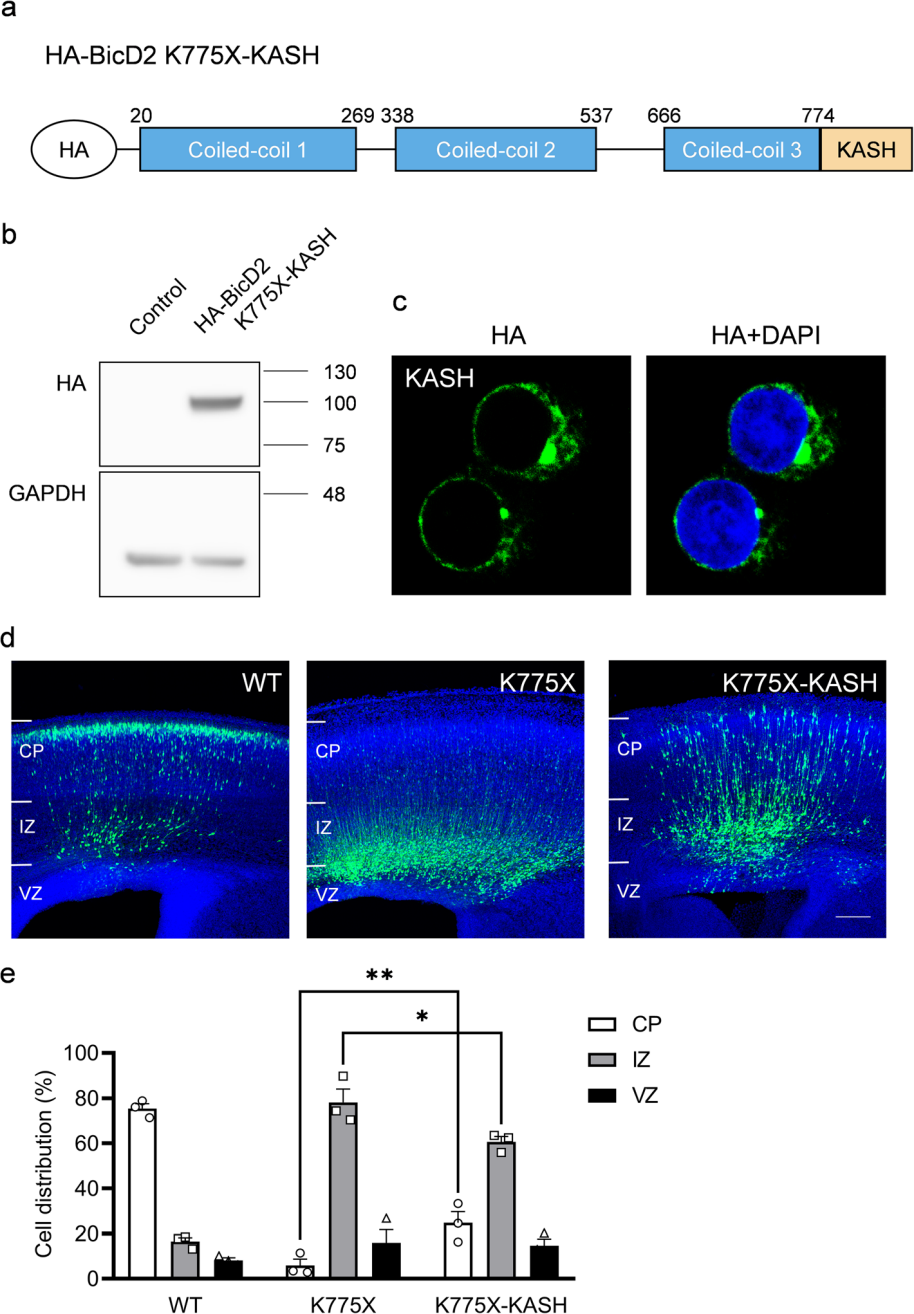
Rescue of neuronal migration defects by the recruitment of BicD2 K775X to the NE. **a** Schematic diagram of the HA-BicD2 K775X protein fused with KASH domain. **b** Expression of HA-BicD2 K775X-KASH in HEK293T cells transfected with the construct. **c** Subcellular localization of HA-BicD2 K775X-KASH (green) in cultured HeLa cells. The protein showed prominent NE localization. Cells were stained with DAPI (blue) to show the cell nuclei. Bar = 5 μm. **d** Coronal sections of mouse brains collected 4 days after electroporation of BicD2 WT, K775X and K775X-KASH at E14.5. Fusion of the KASH domain to BicD2-K775X rescued neuronal migration defects caused by BicD2 K775X mutation. All slices were stained with DAPI (blue) to show the cell nuclei. Bar = 100 μm. **e** Bar graph with individual data points showing cell distribution in the CP, IZ, and VZ 4 days after electroporation (*n* = 3 pregnant females in each condition). Error bars represent SEM. *P < 0.05, **P < 0.01. ANOVA. Post hoc: Bonferroni test

**Fig. 7:**
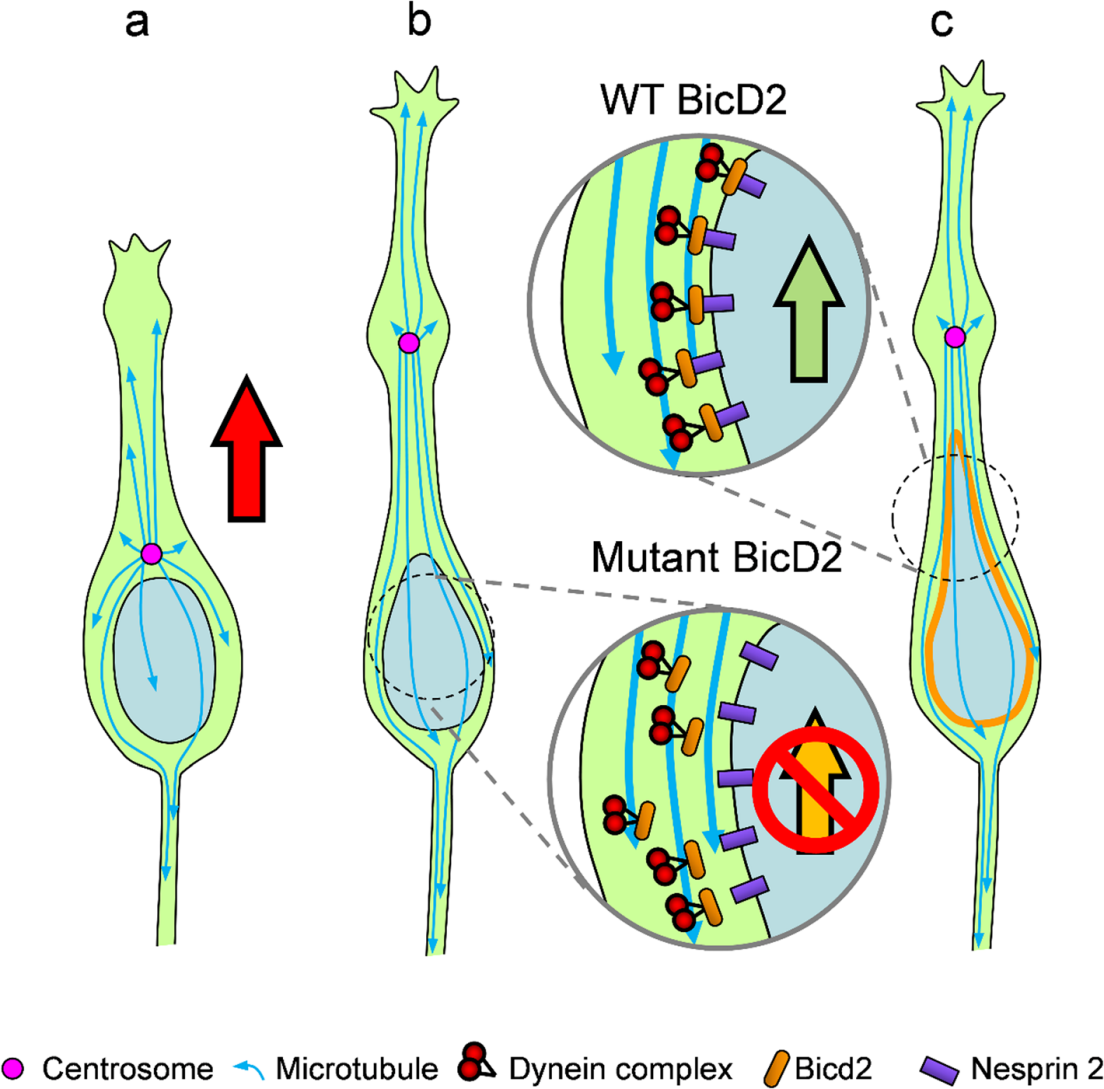
Schematic diagram depicting the mechanism of BicD2 mutation in neuronal migration defects. As cortical neuron migrates, a swelling first forms within the leading process (**a**). The centrosome and the entire microtubule network move into the process by dynein pulling on the plus ends of microtubules (**b**). In the normal condition, BicD2 binds to the NE protein Nesprin-2 and recruits dynein to the nuclear surface. Dynein then engages the plus ends of trailing microtubules and pulls the nucleus forward (**c**). In BicD2 p.K775X mutant cells, the nuclear recruitment is impaired, resulting in failure of dynein NE localization. This defect prohibits the forward movements of the nucleus in the migrating neuron (**b**)

To examine whether the failure of BicD2 K775X recruitment to the NE was the main cause for migration defect in vivo, we fused HA-BicD2 K775X with a KASH domain (K775X-KASH) (Fig. 6a, b), which has previously been shown to direct protein localization to the NE [43]. Expression of K775X-KASH in transfected culture cells showed its prominent localization on the NE (Fig. 6c). We then electroporated the construct into the brain of E14.5 mouse embryos and found that the expression of K775X-KASH partially rescued the neuronal migration delay that resulted from K775X expression (Fig. 6d, e). These results indicated that blocking the recruitment of BicD2 to the NE underlied neuronal migration defects during cortical development (Fig. 7).

## Discussion

In this study, we identified the first de novo *BICD2* nonsense variant p.(Lys775Ter) in a patient presented with posterior predominant lissencephaly and SBH (Fig. 1). In utero electroporation of *BicD2* variants into the developing mouse cortex revealed that expression of BicD2 K775X, but not SMALED2-associated missense variations, causes a dramatic arrest of radial migration in cortical neurons (Figs. 2, 3 and 4). The expression of the K775X mutant also interfered with the NE recruitment of BicD2 and the dynein complex in cultured cells. This C-terminal truncation disrupted the interaction between BicD2 and the nuclear membrane protein Nesprin-2 (Fig. 5). Remarkably, forcing the NE localization of BicD2 K775X by fusion with a C-terminal KASH domain partially rescued neuronal migration defect, further supporting the importance of BicD2 localization to the NE in neuronal migration (Fig. 6). Our results uncover a novel mechanism of lissencephaly by defects in nuclear translocation (but not centrosome movement) during neuronal migration resulting from BICD2 truncated variant (Fig. 7).

### *BICD2* variants in human diseases

Previously, missense variants in human *BICD2* have been found to cause autosomal dominant SMALED2. Studies showed that these variants affect dynein-dynactin-mediated retrograde transport, Golgi fragmentation in cultured cells, microtubule stability, and dynein-dynactin processivity [18, 23, 29, 31, 35]. Motor neurons overexpressing SMALED2-causing *BICD2* variants develop axonal aberrations, such as collateral long branching and overgrowth [29]. Interestingly, in *Drosophila*, neuronal but not muscular expression of BicD mutants that correspond to SMALED2-associated variants led to reduced neuromuscular junction size in larvae and impaired locomotion of adult flies [29], suggesting the neuronal origin of SMA phenotypes in human patients [31, 34, 35]. However, recent genetic experiments in conditional knockout mice suggested that, in SMALED due to BICD2 mutations, motor neuron loss is a non-cell autonomous process arising in muscle. This non-cell autonomous neuronal loss may also contribute to human pathology [37].

Interestingly, missense *BICD2* variant p.Arg694Cys has been reported in two patients of *arthrogryposis multiplex congenita* and cortical malformations, including microcephaly and bilateral perisylvian polymicrogyria [36]. The missense variant p.Arg694Cys is also located in the CC3 domain of BICD2 that binds to Nesprin-2. These findings implicated the important roles of BICD2 in cortical development in humans.

Here we identified the first disease-causing truncated *BICD2* pathogenic variant, which led to SMALED2 and lissencephaly, further supporting the role of BICD2 in human brain development. The major phenotype for this patient was posterior predominant lissencephaly and SBH, which resembles those caused by variants in *LIS1*. LIS1 interacts with the dynein/dynactin complex and BICD2 to recruit cellular structures [42]. Taken together, this suggests that the defects from BICD2 truncation happen mainly in the post-mitotic neuronal migration stage. In our patient, the brain also showed slight hydrocephalus and microcephaly, also consistent with potential roles of BICD2 in earlier stages [24].

### Cellular roles of dynein and BICD2 in brain development

During neuronal migration, the dynein-dynactin complex specifically concentrates at the NE [48], suggesting its potential role in transporting the nucleus as a large cargo [54]. Dynein has later been shown to be essential for the apical movements of the nucleus in radial glia cells undergoing interkinetic nuclear migration [50]. The recruitment of dynein-dynactin complex to the NE is mediated by RabBP2-BicD2 and Nup133-CENP-F pathways during G2 phase [4, 22]. BicD2 knockout mice showed hydrocephaly, thin cortex, and abnormal lamination, suggesting the additional roles of BicD2 in patterning and neurogenesis during early brain development [24]. Strikingly, neuron-specific conditional knockout as well as knockdown of BicD2 in embryonic rodent brains also inhibited the subsequent neuronal migration [22, 56], implicating its roles in neuronal migration at a later stage.

Interestingly, neuronal migration was severely inhibited by not only BicD2 knockdown but also by the expression of the truncated variant K775X. The BICD2 protein mainly consists of coiled-coil (CC) segments 1, 2 and 3, each playing crucial roles in binding to other partners during various functions. The N-terminal CC1 segment binds to the dynein-dynactin complex, CC2 to kinesin KIF5A, and the C-terminal CC3 to several cargo proteins, such as RAB6A, RANBP2 [44], and the newly identified Nesprin-2 [15]. The truncation of the C-terminus in the CC3 domain thus appears to interfere with the ability of BICD2 to bind cargo proteins, as indicated in our results. Previously, a transgenic mouse that overexpressed a CC3-lacking GFP-BICD2-N construct specifically in postnatal motor neurons showed impaired dynein/dynactin functions [46]. However, the influence of the K775X mutant on other protein binding remains to be determined.

### Involvement of Nesprin-1/2 in neural development

In the current study, we showed that the interaction between BicD2 and Nesprin-2 is disrupted by the lissencephaly-associated p.(Lys775Ter) variant. Nesprin-2 contains a conserved KASH domain at the C-terminus, which targets the outer nuclear membrane by interacting with SUN proteins. The N-terminus of Nepsrin2 contains a CH domain, which binds to actin, and a variable number of spectrin repeats (SR), which interact with cytoskeletal and motor proteins [57]. Previously, Nesprin-1/2 double knockout mice showed severe lamination defects in the cerebral cortex. Nesprin-2 KO alone is sufficient for the lamination in the cerebral cortex, further supporting that Nesprin-2 plays an essential role in the formation of cerebral cortex structure [58]. While the current study focused on dynein in nuclear translocation during neuronal migration, it is possible that BicD2 could also regulate or be regulated by actin dynamics during the neuronal migration cycle, given that Nesprins bind actin and can accumulate at the front of the nucleus during migration [10].

### Potential dominant-negative effects of BICD2 mutations

Interestingly, we also found that the expression of BicD2 K775X alone appeared to cause more severe impairment of neuronal migration, while expression of WT or other missense variants did not have apparent effects on neuronal migration. Furthermore, this truncated BicD2 mutant failed to localize to the NE, and hinders NE localization of dynein-dynactin complex. We therefore suspected that BicD2 K775X expression may have a dominant-negative effect on neuronal migration by trapping dynein-dynactin in the cytoplasm. Importantly, the heterozygote knockout mouse did not exhibit an abnormal phenotype in the nervous system [24], further supporting that the phenotype results from a dominant-negative effect rather than haploinsufficiency.

So far, all of the reported SMALED2-causing variants were heterozygous missense changes [23, 28, 29]. To date, neither frameshift variant, nonsense variant, nor gene deletions of BICD2 have been reported in association with SMALED2. This suggests that loss-of-function variants of BICD2 are not responsible for SMALED2, and that the pathophysiological mechanism of non-truncating variants of *BICD2* has rather a dominant-negative or a gain-of-function effect [47]. This hypothesis is compatible with the several mechanisms proposed to explain the pathogenicity of BICD2 variants. It has been shown that pathogenic BICD2 variants can lead to hyperactivated dynein-dynactin complex motility, and thus to an imbalance between anterograde and retrograde transport [23]. The truncated variant reported in this study is probably unique as it is on the penultimate exon of *BICD2* gene, which is predicted to escape the translation-dependent nonsense mediated mRNA decay [27]. Hence, the resulting defective protein may trap dynein and exert a dominant negative effect due to its inability to localize to the NE through interaction with the NE protein Nesprin-2.

In conclusion, we identified a novel de novo truncated *BICD2* variant in a posterior predominant lissencephaly patient. Functional assays showed that this particular variation caused neuronal migration defects during development. Impairment of nuclear translocation but not centrosome movement is a key pathomechanism of human neuronal migration disorders. Our results also widen the phenotypical spectrum of *BICD2* variants in humans and illustrated the importance of dynein recruitment to the NE during neuronal migration.

## Materials and methods

### Study subjects

The proband was referred to the genetic study program at the Department of Neurology at Kaohsiung Chang Gung Memorial Hospital, Taiwan. Another 63 healthy Taiwanese individuals without epilepsy or known neurological disorders from our previous studies were included as controls. Clinical phenotyping was performed by direct interviews as well as reviews of medical records and neuroimaging studies. The study was approved by the local human research ethic committee (201601359B0) and written consent was obtained from the parents.

### Whole exome sequencing

WES was performed as previously described [53]. In brief, genome DNA was captured using Agilent SureSelect Human All Exon V6 and sequenced using Illumina NovaSeq system (WELGENE Biotech, Taiwan). Reads were analyzed by standardized bioinformatics pipeline, variants were called using FreeBayes and annotated with ANNOVAR as detailed in previous study [52]. Variants were then filtered using the following criteria: read depth > 20, located in exon/splice site, not synonymous, not presented in dbSNP, the Thousand Genome Project, the ExAC or the genomAD database. We also filtered the variants using in-house 63 Taiwanese healthy control WES. Only loss of function variants or missense variants predicted to be pathogenic by all four in silico programs (PolyPhen2, PROVEAN, MutationTaster2 and CADD≥20) were validated with Sanger sequencing in the proband and the unaffected parents.

### Knock-down constructs

For RNA interference, BicD2 short hairpin RNA (shRNA) constructs in a lentiviral vector (pLKO.1-puro) were purchased from RNAi Core of Academia Sinica (Taiwan. Both shBicD2 target sequences (shBicD2–3’U: 5′-TGAGT AGTAT TACCT ACAAAT-3′; shBicD2-CDS: 5′-GCCAA CCTGA AGAGC AAGTAT-3′) were chosen to target 3’UTR and CDS of BicD2 mRNA respectively. The control shRNA (shCtrl: 5′-CCTAA GGTTA AGTCG CCCTCG-3′) does not affect the expression of BicD2 (Fig. 1a).

### Plasmids and antibodies

The mouse BicD2 cDNA construct was purchased from Sino Biological (MG5A2126-NY, GenBank Accession No.: NM_001039179.2) and was cloned to pCAGGS construct, which was a gift from Phil Sharp (Addgene plasmid # 41583) [17] with an HA tag. US2-GFP construct was a generous gift from Dr. Jenn-Yah Yu (National Yang-Ming University Taiwan). The sources of the primary antibodies used in this study are listed as follows: Rabbit anti-Ki67 (AB9260, Millipore), Rabbit anti-Pax-6 (#901301, BioLegend), anti-Cux1 antibody (sc-13,024, Santa Cruz), Rabbit anti-NeuN (ABN78, Millipore), Rabbit anti-BICD2 (ab117818, Abcam), Rabbit anti-HA (GTX115044, Genetex), Mouse anti-HA (66006–1-Ig, Proteintech), Mouse anti β-actin (66009–1-Ig, Proteintech), Mouse anit-α-tubulin (66031–1-Ig, Proteintech), Mouse anti-lamin A/C 636 (sc-7292, Santa Cruz), Mouse anti-Dynein heavy chain C-5 (sc-514,579, Santa Cruz), Mouse anti-Dynein light intermediated chain clone 74.1 (MAB1618, Millipore).

### Western blot analysis

To examine the knockdown efficiency of shBicD2 shRNA, shRNA constructs were packaged to lentiviral particles and delivered into primary cultured cortical neurons by lentiviral infection [32]. After puromycin selection for 48 h, cells were lysed in RIPA buffer (Sigma Aldrich) supplemented with a protease inhibitor cocktail (Roche) and the protein concentration was determined by BCA protein assay (Thermo Fisher Scientific). Expression of BicD2 protein was identified using western blotting. Beta-actin was used as the internal control. Protein bands on the PVDF membrane (Millipore) were visualized by ECL reagent (Millipore) and detected by Luminescence Imaging system LAS-4000 (Fujifilm). The images were quantified using ImageJ software (National Institutes of Health, NIH, USA).

### In utero electroporation

All experiments and animal maintenance were conducted according to protocols approved by the Institutional Animal Care and Use Committee (IACUC) at National Yang-Ming University. In utero electroporation of embryos in pregnant ICR mice was conducted as previously described [26, 38, 45, 49]. In brief, solution containing 1.5 μg/ul of shRNA construct or 0.5 μg/ul of overexpression of cDNA construct together with 0.5 μg/ul of US2-GFP plasmid were injected into the lateral ventricle of embryonic brains at E14.5 and electroporated with a forceps electrode, which transmitted five electric pulses at 40 V for 50 ms at 1-s interval through the uterine wall. The embryos were harvested at the indicated time after the surgery or birth and then the brains were transcardially perfused with PBS and subsequently 4% Paraformaldehyde (PFA) for tissue fixation.

### Live brain slice imaging

The live brain slice imaging was performed as previous described [8, 9, 51]. Briefly, live mouse brains were coronal sectioned into 350 μm-thick slices using the Vibratome (Leica) 2.5 days after electroporation. Then, the slices were placed on Millicell-CM inserts (Millipore) in culture medium containing 25% Hanks balanced salt solution, 47% basal MEM, 25% normal horse serum, 1% penicillin/streptomycin/glutamine (GIBCO BRL), and 0.66% glucose. GFP positive cells were imaged by inverted fluorescent microscope or LSM880 confocal microscope (Carl Zeiss), while incubated at 37 °C in 5% CO_2_. The time-lapse images were captured at 5 min interval for 220 cycles or 10 min interval for 50 cycles.

### Immunofluorescence staining

Brain slices were washed with PBS, followed by PBST (0.2% of Triton X-100 in PBS) permeabilization for 30 min. The slices were then incubated in blocking buffer (10% NGS + 5% BSA in PBST) for 1 h, then treated with primary antibodies dissolved in PBST containing 5% NGS and 5% BSA for 2 days. After they were washed with PBS, slices were incubated in fluorescent-dye conjugated secondary antibodies (Alexa Fluor™ 546 goat anti-rabbit IgG, Thermo Fisher Scientific) with the indicated wavelength for 2 h. Finally, slices were counterstained with DAPI (Thermo Fisher Scientific) for 1 h and mounted using antifade mounting medium (H-1000, Vector Laboratories).

### Nucleus-centrosome coupling assay

To label the centrosome in neurons, Cent2-DsRed cDNA was subcloned into a pCAGIG construct, which was a gift from Connie Cepko (Addgene plasmid # 11159), termed as pCAGIG-Cent2-DsRed. In utero electroporation was performed at E14.5 to deliver pCAGIG-Cent2-DsRed into neural stem cells and the embryos were harvested at E17.5. The brain slices were stained with DAPI (Thermo Fisher Scientific) to show the cell nuclei and imaged with an inverted laser scanning confocal microscope (Zeiss LSM880) with a 40X Plan-Apochromat NA = 1.3 oil objective. Image condition is described in the Microscopy part. ImageJ software was used to measure the nucleus-centrosome distance.

### Cell culture, transient transfection and immunocytochemistry

HEK293T, HeLa and U2OS cell lines were used in this study. The HeLa and U2OS cells were gifts from Prof. Tzu-Hao Cheng and Prof. Won-Jing Wang, respectively (Institute of Biochemistry and Molecular Biology, National Yang-Ming University). All cell lines were cultured in Dulbecco’s Modified Eagle Medium (DMEM) with 10% fetal bovine serum (FBS) and 1% penicillin-streptomycin (Thermo Fisher Scientific) and maintained in a humidified atmosphere of 5% CO_2_ at 37 °C. Transient transfection was carried out by Lipofectamine 3000 (Thermo Fisher Scientific) according to the manufacturer’s instructions.

HeLa and U2OS cells were plated on 12-mm-diameter glass coverslips coated with poly-D-lysine (Sigma Aldrich) for cell staining [21, 25]. HA-BicD2 WT or other mutant constructs were transfected into cells for 24 h. For identifying the interaction between Nesprin-2 and BicD2, cells were synchronized in G0 phase by serum starvation for 24 h. The detailed steps for cell staining were performed as described previously [22].

### Primary cortical neuronal culture

Cortical neuronal culture was prepared from the neocortex of E17.5 mice co-electroporated with HA-BicD2 WT or K775X cDNA along with pCAG-GFP construct at E14.5. Tissues were dissociated with neuron dissociation solutions according to the manufacturer’s instruction (FUJIFILM). Neurons were cultured in Neurobasal Medium supplemented with B27 and GlutaMax (Thermo Fisher Scientific) and plated on the cover glass coated with 0.1 mg/ml of poly-D-lysine (Sigma-Aldrich) and 30X-diluted matrigel (Corning). Cells were fixed with 4% PFA at day in vitro (DIV) 7 for subsequent immunofluorescence staining.

### Microscopy

All the fixed brain slices were imaged with an inverted laser scanning confocal microscope (Zeiss LSM880) with a 20X Plan-Apochromat NA = 0.8 objective. The DAPI signal was excited by a 405 nm Diode laser; green fluorescence signal (GFP or Alexa 488) was excited by a 488 nm Argon laser; red fluorescence signal (Cent2-DsRed or Alexa 546) was excited by a 555 nm HeNe laser and infrared fluorescence signal was excited by a 633 nm HeNe laser [7].

Cells were imaged using a confocal microscope (Zeiss LSM700) with a 100X Plan-Apochromat NA = 1.4 oil objective. Alexa Fluor 488 was excited by a 488-nm laser. Alexa Fluor 546 was excited by a 555-nm laser. Signal of DAPI was excited by a 405-nm laser. Analysis of images were carried out with Zen software (Zeiss) of ImageJ (NIH) [21, 25].

### GST-pull down assay

Nesprin-2 C-terminus includes Spectrin 54–56 (NM_001005510.2, aa 6343–6767) which was amplified from mouse kidney cDNA and sub-cloned into a pGEX4T-1 construct. Those constructs were transformed into E.*coli* strain BL21(DE3). The colonies were inoculated into 5 ml of LB broth with Ampicillin at 37 °C overnight, which was then diluted 50-fold in 50 ml of LB broth and grown at 37 °C until the number of bacteria increased to the proper concentration by measuring the OD600 to 0.4. 0.2 mM of IPTG (Sigma Aldrich) was used to induce the expression of GST proteins at 30 °C for 3 h. Bacterial lysates were harvested and lysed by B-PER bacterial protein extraction reagent (Thermo Fisher Scientific), and then incubated with 0.5 ml of Glutathione Sepharose 4B resin (GE Healthcare) for 1 h at 4 °C. The concentration of purified Nesprin-2 C-terminus was determined by SDS-PAGE. HEK293T cells transfected with pCAGGS-HA-BicD2 WT or K775X constructs were harvested in RIPA buffer and incubated with glutathione resins conjugated with Nesrin2 C-terminus overnight at 4 °C. Western blotting was applied to quantify the interaction between BicD2 and Nesprin-2 C-terminus.

### Statistical analysis

Statistical analysis was performed using GraphPad Prism (version 8.0) and Excel (Microsoft). *p*-value < 0.05 was assumed as statistically significant. All statistical details such as types of statistical test utilized, number of biological replicates performed, number of cells counted, number of animals used, and meanings of error bars are described in Figure Legends.

## Supplementary information

**Additional file 1: Figure S1.** BicD2 expression and cell distributions in the developing cortex subjected to BicD2 RNAi. **Figure S2.** Immunostaining of different markers in brain slices electroporated with Bicd2 K775X.

**Additional file 2: Movie S1.** Wildtype BicD2 expressing neural progenitor in IZ perform a normal basally directed radial migration at E16.5. The time format is mm:ss. Scale bar = 5 μm.

**Additional file 3: Movie S2.** Mutant (K775X) BicD2 expressing neural progenitor in IZ show a totally disrupted radial migration at E16.5. The time format is mm:ss. Scale bar = 5 μm.

## Data Availability

All data associated with this study are available in the main text and supplementary information. Materials are available from the corresponding author upon reasonable request.
