# Supplementary material for: Impairment in dynein-mediated nuclear translocation by BICD2 C-terminal truncation leads to neuronal migration defect and human brain malformation
Source: Acta Neuropathol Commun. 2020 Jul 14;8:106. doi: 10.1186/s40478-020-00971-0 (PMC7362644; doi:10.1186/s40478-020-00971-0)
Supplement: Supplementary file 1 — Additional file 1: Figure S1. BicD2 expression and cell distributions in the developing cortex subjected to BicD2 RNAi. Figure S2. Immunostaining of different markers in brain slices electroporated with Bicd2 K775X. [file 40478_2020_971_MOESM1_ESM.pdf]

SUPPLEMENTARY MATERIALS:

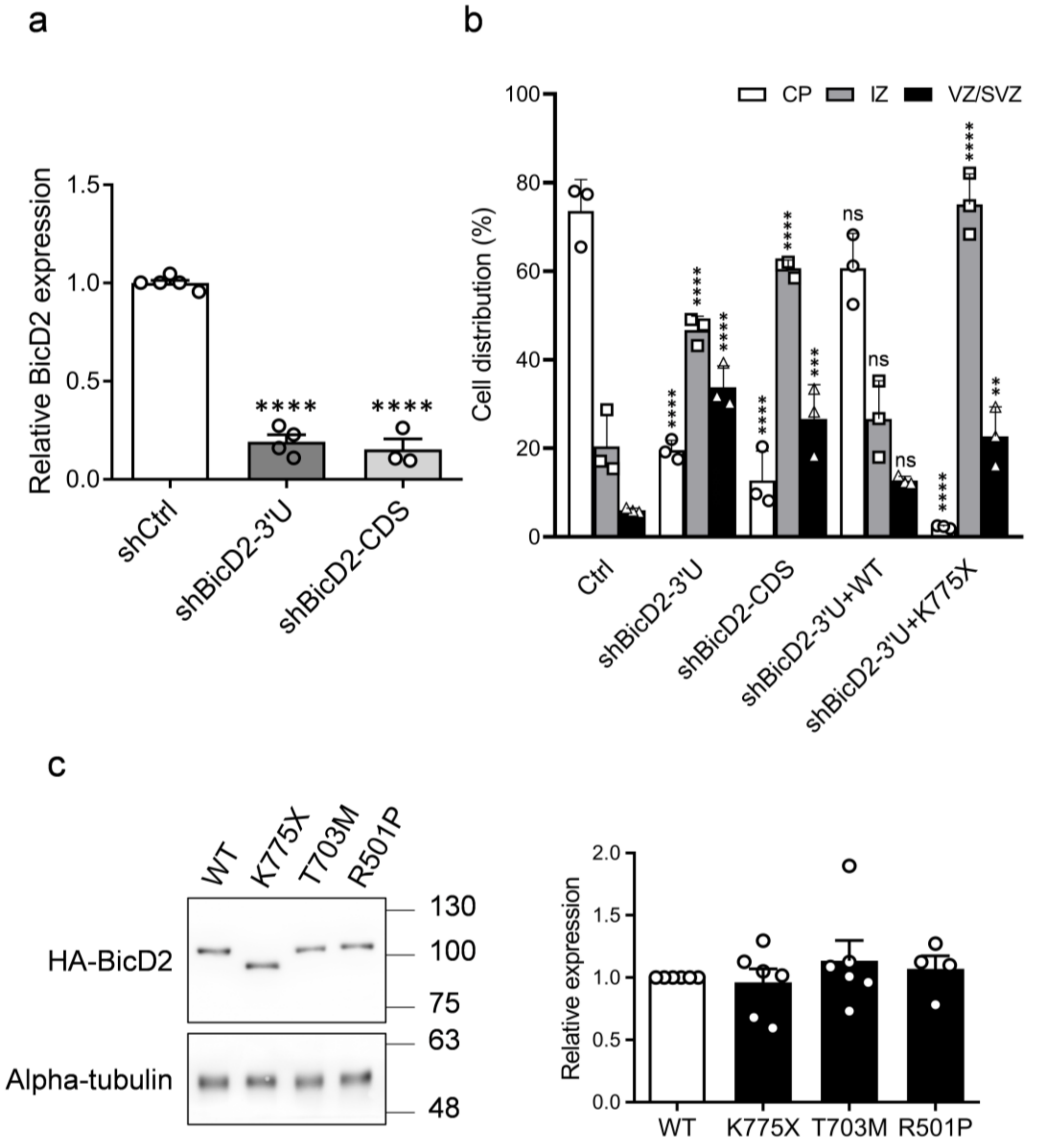

Figure S1. BicD2 expression and cell distributions in the developing cortex subjected to BicD2 RNAi. (A) BicD2 expression in cultured mouse neurons infected with lentiviruses encoding shCtrl or shRNAs targeting the 3’UTR and CDS of BicD2 ($n \geq 3$ independent experiments). Error bars represent SEM., ****$P < 0.0001$. ANOVA test. Post hoc: Bonferroni test. (B) Bar graph with individual data points showing altered cell distributions corresponding to Figure 2B. ($n = 3$ pregnant females in each condition). Error bars represent SEM., **: $p < 0.01$, ***$P < 0.001$, ****$P < 0.0001$. ANOVA test. Post hoc: Bonferroni test. (C) Expression of WT, K775X, T703M, and R501P variants of BicD2 in cultured cells. There was no significant difference between different variants. ANOVA test ($n \geq 3$ independent experiments).

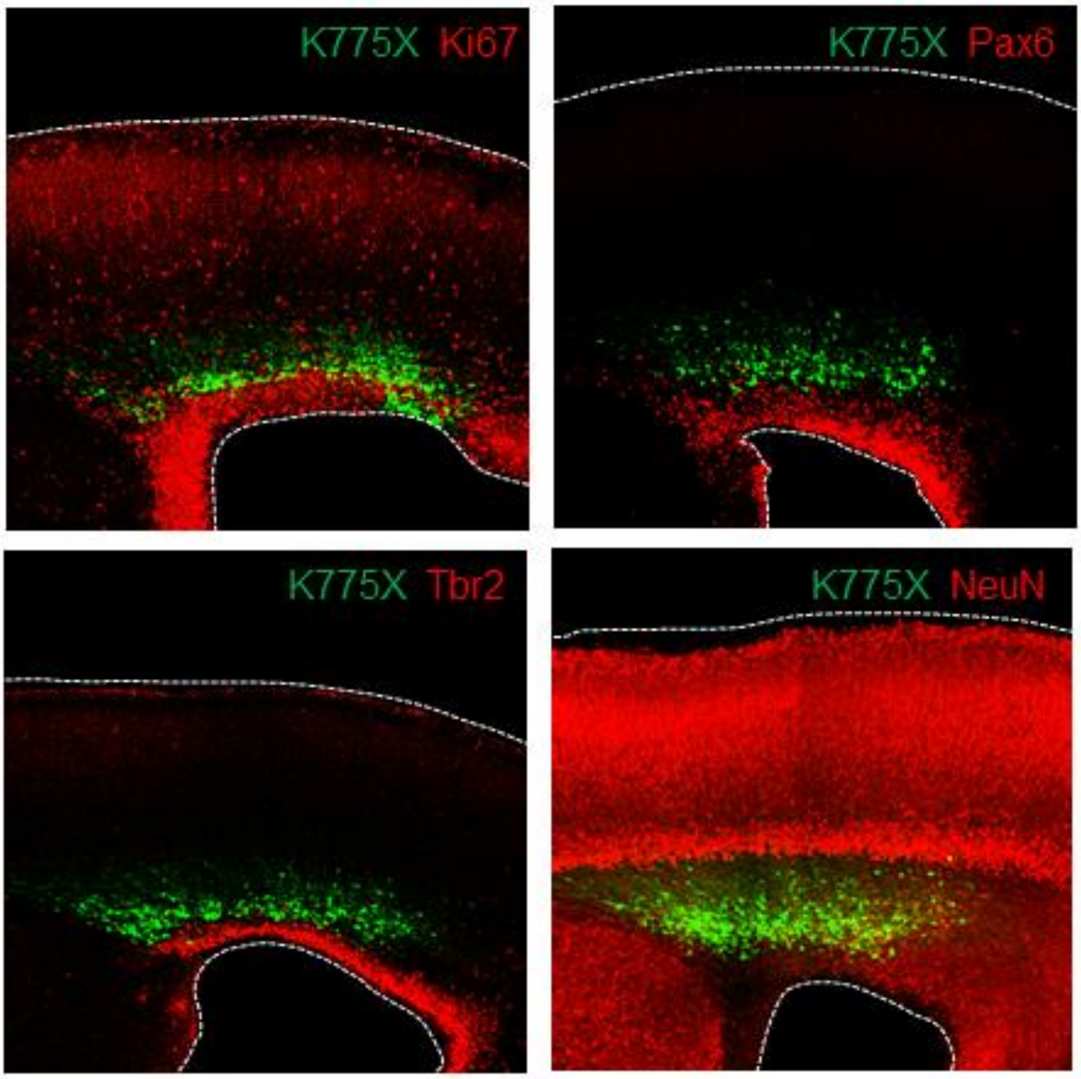

Figure S2. Immunostaining of different markers in brain slices electroporated with BicD2 K775X. Brains were electroporated at E14.5 and stained with the cell cycle marker Ki67, neural progenitor maker Pax6, intermediate progenitor marker Tbr2, and mature neuronal marker NeuN (red) at E18.5. Virtually all of the K775X-electroporated cells (green) were negative to these markers at this stage.
